# Effect of Flavonoids on MCP-1 Expression in Human Coronary Artery Endothelial Cells and Impact on MCP-1-Dependent Migration of Human Monocytes

**DOI:** 10.3390/ijms242216047

**Published:** 2023-11-07

**Authors:** Lea Brüser, Elisa Teichmann, Burkhard Hinz

**Affiliations:** Institute of Pharmacology and Toxicology, Rostock University Medical Center, Schillingallee 70, 18057 Rostock, Germany; lea.brueser@uni-rostock.de (L.B.); elisa.teichmann@web.de (E.T.)

**Keywords:** monocyte chemoattractant protein-1, flavonoids, vascular endothelial cells, monocyte migration

## Abstract

The monocyte chemoattractant protein-1 (MCP-1), also known as chemokine (CC motif) ligand 2 (CCL2), is involved in the formation, progression, and destabilization of atheromatous plaques. Flavonoids, found in fruits and vegetables, have been associated with various health-promoting properties, including antioxidant, anti-inflammatory, and cardioprotective effects. In the present study, the flavonoids quercetin, kaempferol, and luteolin, but not cannflavin A, were shown to substantially inhibit interleukin (IL)-1β-induced MCP-1 mRNA and protein expression in human coronary artery endothelial cells (HCAEC). At the functional level, conditioned medium (CM) from IL-1β-stimulated HCAEC caused an increase in the migration of THP-1 monocytes compared with CM from unstimulated HCAEC. However, this induction was suppressed when IL-1β-treated HCAEC were coincubated with quercetin, kaempferol, or luteolin. The functional importance of MCP-1 in IL-1β-induced monocyte migration was supported by experiments showing that neutralization of MCP-1 in the CM of IL-1β-treated HCAEC led to a significant inhibition of migration. In addition, a concentration-dependent induction of monocyte migration in the presence of recombinant MCP-1 was demonstrated. Collectively, the flavonoids quercetin, kaempferol, and luteolin were found to exert potential antiatherogenic effects in HCAEC, challenging further studies with these compounds.

## 1. Introduction

Cardiovascular diseases, including atherosclerosis, are the leading cause of death worldwide [1]. A key factor in cardiovascular diseases, particularly in the development of atherosclerosis, is inflammation [2], with vascular endothelial cells having an important role in this process [3,4]. Chemokines that influence the progression of atherosclerosis by recruiting leukocytes are a major player in vascular inflammation (for review, see [5]). Monocyte chemoattractant protein-1 (MCP-1), also known as chemokine (CC motif) ligand 2 (CCL2) (for review, see [6,7,8]), is one such proinflammatory chemokine that primarily mediates the migration of monocytes to sites of inflammation [9]. Thereby, the attracted monocytes attach to the activated endothelium, migrate to the subendothelial tissue and subsequently differentiate into macrophages. MCP-1, which consists of 76 amino acids with a molecular mass of approximately 13 kDa [10], is expressed by various cell types such as monocytes, smooth muscle cells, and vascular endothelial cells in response to proinflammatory stimuli like interleukin (IL)-1β and tumor necrosis factor (TNF)-α [5,6,7,8]. The main receptor targeted by MCP-1 is CC chemokine receptor 2 (CCR2), which is primarily located on immune cells such as monocytes, macrophages, and activated T cells [5,6,7,8]. MCP-1 is highly expressed in human atherosclerotic lesions [11] and targeting MCP-1 or its receptor CCR2 reduces the burden of atherosclerotic lesions and decreases macrophage accumulation [12]. In line with this notion, a strategy to use anti-MCP-1 gene therapy to reduce atherogenesis has been successfully investigated in apolipoprotein E knockout mice [13]. In a recent analysis, higher MCP-1 plaque levels were associated with histopathological, molecular, and clinical hallmarks of plaque vulnerability in patients undergoing carotid endarterectomy [14]. Finally, a meta-analysis of seven cohort studies found that higher circulating MCP-1 levels were associated with higher long-term cardiovascular mortality in community-dwelling individuals without apparent cardiovascular disease [15]. Overall, lowering MCP-1, a key regulator of monocyte transport, is therefore considered a promising strategy to combat cardiovascular disease [16].

**Figure 1:**
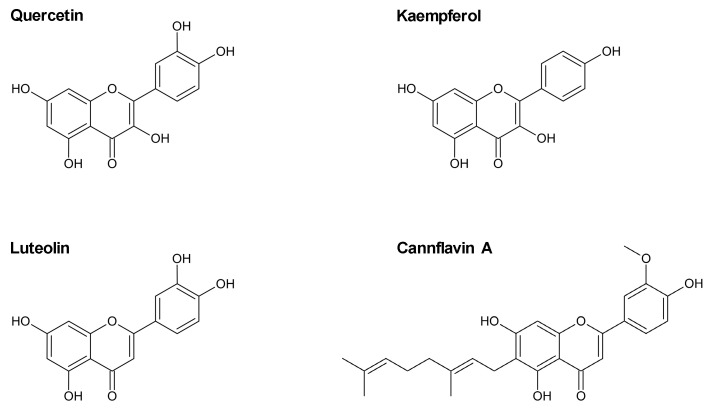
Chemical structures of the flavonoles quercetin and kaempferol and the flavones luteolin and cannflavin A.

Flavonoids, secondary metabolites found mainly in fruits and vegetables, are associated with a variety of health-promoting effects [17]. According to epidemiological studies, a positive association between diets high in flavonoid-rich foods and cardiovascular health has been suggested (for review, see [18,19]). Flavonoids are classified according to their structure, with flavonols, flavones, flavanols, flavanones, anthocyanidins, isoflavones, and chalcones among the most important groups [20]. Flavonols, especially quercetin and kaempferol (Figure 1), are considered the most abundant flavonoids found in foods [21]. Quercetin, in particular, has gained attention in recent years as a potential pharmaceutical agent for the treatment of cardiovascular diseases [22]. In addition, the flavone luteolin (Figure 1) has been shown to exhibit cardioprotective effects in mouse models of cardiomyopathy and myocardial injury [23,24]. Another flavone, cannflavin A (for review, see [25]; Figure 1), isolated from *Cannabis sativa* L., has not yet been studied in endothelial cells.

Although the reduction in MCP-1 levels by various flavonoids has been demonstrated in a number of cell types, including endothelial cells, to our knowledge, no corresponding studies have been performed in human coronary artery endothelial cells (HCAEC). On the other hand, previous studies have already identified a number of protective effects of flavonoids on HCAEC such as inhibition of the endothelial–mesenchymal transition [26], suppression of proinflammatory nuclear factor kappa B (NF-κB) signaling [27,28], activation of endothelial nitric oxide (NO) synthase [29,30], or downregulation of plasminogen activator inhibitor-1 (PAI-1) expression [31]. Consequently, identifying additional targets is a challenge.

Using HCAEC, the present study therefore investigates the effect of four different flavonoids, namely quercetin, kaempferol, luteolin, and cannflavin A, on the expression of the proinflammatory chemokine MCP-1. Here, we show that quercetin, kaempferol, and luteolin, but not cannflavin A, substantially inhibit IL-1β-induced mRNA expression and secretion of the chemokine MCP-1 in HCAEC, thereby also interfering with monocyte migration toward the conditioned medium (CM) of IL-1β-stimulated HCAEC.

## 2. Results

### 2.1. Flavonoids Do Not Impair the Viability of HCAEC at Low Concentrations

**Figure 2:**
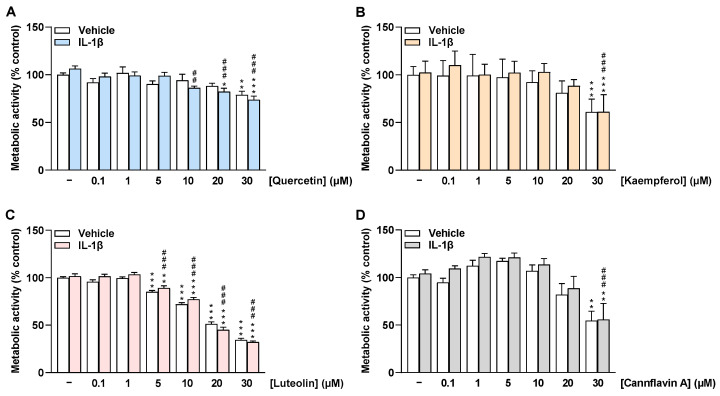
Effect of flavonoids on metabolic activity of HCAEC under basal or IL-1β-stimulated conditions as determined by the MTT assay. Cells were preincubated with increasing concentrations of quercetin (**A**), kaempferol (**B**), luteolin (**C**), cannflavin A (**D**), or vehicle for 1 h, followed by the addition of 10 ng/mL IL-1β or its vehicle and subsequent coincubation for 24 h. Thereafter, metabolic activity was determined via MTT assay. Viability values of vehicle-treated cells were set to 100%. Data are presented as means ± SEM of *n* = 9 (**A**–**C**) or *n* = 8–9 (**D**) of three independent experiments each. * *p* ≤ 0.05, ** *p* ≤ 0.01, *** *p* ≤ 0.001 vs. vehicle control (leftmost white bar); ## *p* ≤ 0.01, ### *p* ≤ 0.001 vs. IL-1β-stimulated cells; one-way ANOVA with Bonferroni’s post hoc test.

To investigate whether the selected flavonoids affect HCAEC viability under basal and IL-1β-stimulated conditions, MTT assays were performed after 24 h of incubation to determine the metabolic activity of HCAEC. Here, quercetin treatment had no effect on HCAEC metabolic activity under basal and IL-1β-stimulated conditions up to 20 µM and 5 µM, respectively (Figure 2A). The flavone luteolin altered the metabolic activity of HCAEC from 5 µM by about 20% to 30 µM by about 70% (Figure 2C). In contrast, kaempferol and cannflavin A showed no significant change in HCAEC metabolic activity under basal and stimulated conditions up to 20 µM (Figure 2B,D). Furthermore, WST-1 assays were carried out as an additional method to determine the metabolic activity of HCAEC (Supplementary Figure S1), with the results obtained here being similar to those obtained in the MTT assays.

### 2.2. Flavonoids Do Not Impair the Cell Number of HCAEC at Low Concentrations

**Figure 3:**
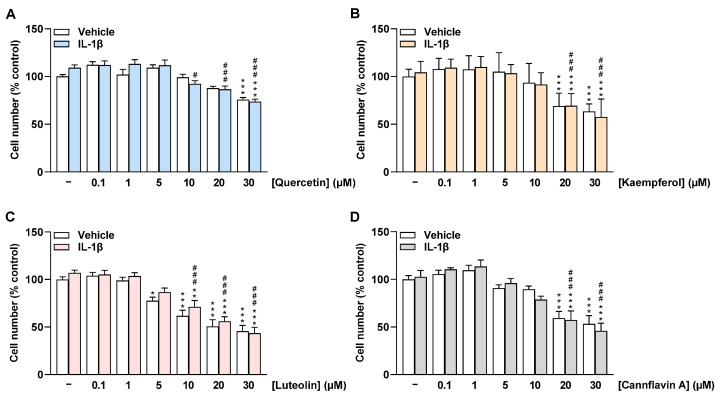
Effect of flavonoids on cell number of HCAEC under basal or IL-1β-stimulated conditions as determined by the crystal violet assay. Cells were preincubated with increasing concentrations of quercetin (**A**), kaempferol (**B**), luteolin (**C**), cannflavin A (**D**), or vehicle for 1 h, followed by the addition of 10 ng/mL IL-1β or its vehicle and subsequent coincubation for 24 h. Thereafter, cell number was determined via crystal violet staining. Cell number values of vehicle-treated cells were set to 100%. Data are presented as means ± SEM of *n* = 9 (**A**,**C**,**D**) or *n* = 8–9 (**B**) of three independent experiments each. * *p* ≤ 0.05, ** *p* ≤ 0.01, *** *p* ≤ 0.001 vs. vehicle control (leftmost white bar); # *p* ≤ 0.05, ### *p* ≤ 0.001 vs. IL-1β-stimulated cells; one-way ANOVA with Bonferroni’s post hoc test.

In addition to HCAEC metabolic activity, the effect of flavonoids on HCAEC cell number under basal and IL-1β-stimulated conditions was determined via crystal violet staining after 24 h of incubation. Similar to the metabolic activity of cells, quercetin did not significantly affect the number of HCAEC treated with vehicle and IL-1β up to 20 µM and 5 µM, respectively (Figure 3A). Luteolin significantly decreased HCAEC cell number from 5 µM under basal conditions and from 10 µM under IL-1β-stimulated conditions (Figure 3C). Kaempferol and cannflavin A were found to significantly reduce HCAEC cell number at concentrations of 20 µM and 30 µM, respectively (Figure 3B,D). In further experiments, accordingly, only concentrations up to 20 µM for quercetin, kaempferol, and cannflavin A and up to 10 µM for luteolin were included.

### 2.3. Quercetin, Kaempferol, and Luteolin Inhibit IL-1β-Induced MCP-1 mRNA Expression in a Concentration-Dependent Manner, Whereas Cannflavin A Increases MCP-1 mRNA Levels in HCAEC

**Figure 4:**
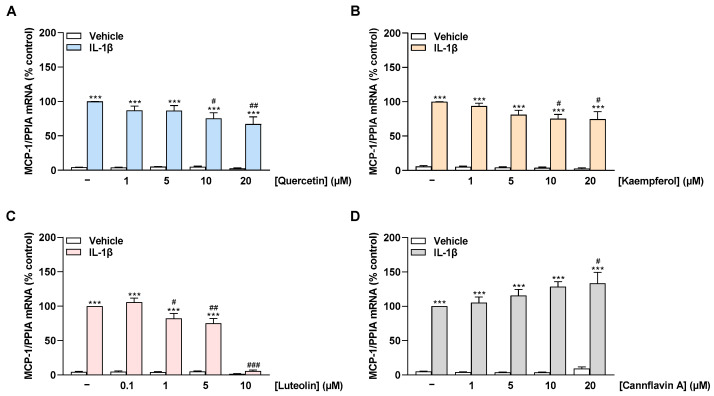
Effect of flavonoids on MCP-1 mRNA expression in HCAEC under basal or IL-1β-induced conditions. Cells were preincubated with increasing concentrations of quercetin (**A**), kaempferol (**B**), luteolin (**C**), cannflavin A (**D**), or vehicle for 1 h, followed by the addition of 10 ng/mL IL-1β or its vehicle and subsequent coincubation for 8 h. Thereafter, mRNA expression was determined via qRT-PCR. PPIA-normalized MCP-1 mRNA values from cells treated with IL-1β and the vehicle of test compounds (first colored bar from the left) were set to 100%. Data are presented as means ± SEM of *n* = 3 (three independent experiments). *** *p* ≤ 0.001 vs. vehicle control (leftmost white bar); # *p* ≤ 0.05, ## *p* ≤ 0.01, ### *p* ≤ 0.001 vs. IL-1β-stimulated cells; one-way ANOVA with Bonferroni’s post hoc test.

To reveal a possible influence of flavonoids on MCP-1, the mRNA levels of the chemokine were examined in non-stimulated as well as IL-1β-stimulated HCAEC treated with the cytokine for 8 h. Stimulation of HCAEC with IL-1β significantly increased MCP-1 mRNA expression compared with vehicle-treated cells, consistent with an inflammatory state in the cells (Figure 4A–D). The flavonols quercetin and kaempferol suppressed IL-1β-induced MCP-1 mRNA expression in a concentration-dependent manner with a significant reduction starting at 10 µM (Figure 4A,B). Luteolin showed a slight reduction in induced MCP-1 mRNA expression at 1 µM and 5 µM and an extreme reduction at 10 µM to basal levels (Figure 4C). In contrast, cannflavin A concentration-dependently increased the IL-1β-stimulated MCP-1 mRNA levels up to 30% at 20 µM (Figure 4D).

### 2.4. Flavonoids Decrease IL-1β-Stimulated MCP-1 Secretion of HCAEC

**Figure 5:**
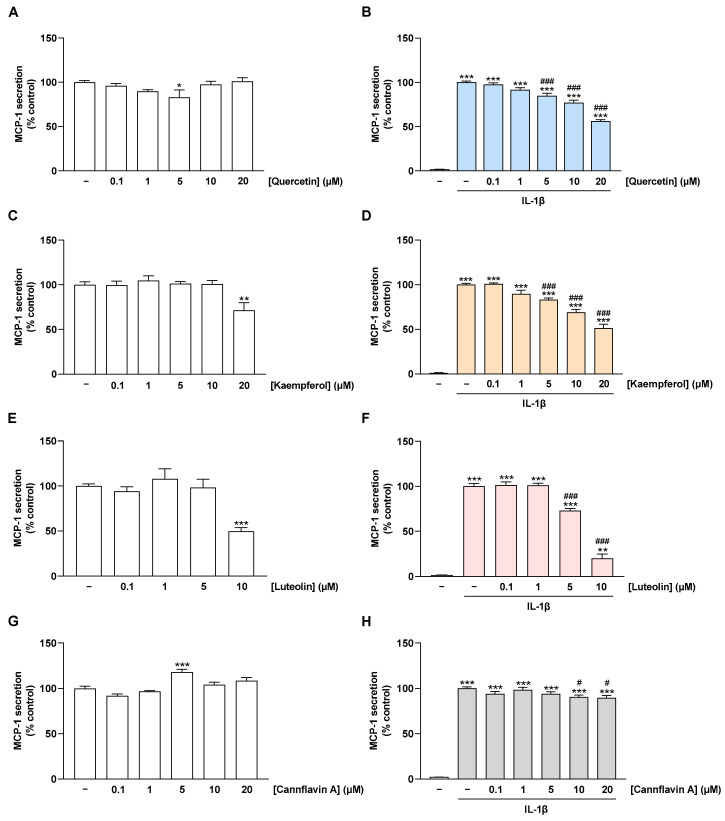
Effect of flavonoids on MCP-1 secretion in HCAEC under basal or IL-1β-induced conditions. In (**B**,**D**,**F**,**H**), cells were preincubated with increasing concentrations of quercetin, kaempferol, luteolin, cannflavin A, or vehicle for 1 h, followed by the addition of 10 ng/mL IL-1β or its vehicle and subsequent coincubation for 24 h. Accordingly, an incubation time of 25 h with flavonoids was chosen for basal conditions (**A**,**C**,**E**,**G**). Thereafter, MCP-1 secretion was determined via MCP-1 ELISA. MCP-1 levels from cells treated with vehicle (**A**,**C**,**E**,**G**) or vehicle in combination with IL-1β (**B**,**D**,**F**,**H**) were set to 100%. Data are presented as means ± SEM of *n* = 9 (three independent experiments). * *p* ≤ 0.05, ** *p* ≤ 0.01, *** *p* ≤ 0.001 vs. vehicle control; one-way ANOVA with Dunnett’s post hoc test (**A**,**C**,**E**,**G**). ** *p* ≤ 0.01, *** *p* ≤ 0.001 vs. vehicle control (leftmost white bar); # *p* ≤ 0.05, ### *p* ≤ 0.001 vs. IL-1β-stimulated cells; one-way ANOVA with Bonferroni’s post hoc test (**B**,**D**,**F**,**H**).

After analysis of MCP-1 mRNA, the effect of flavonoids on MCP-1 protein levels in supernatants of non-stimulated and IL-1β-stimulated HCAEC, hereafter referred to as MCP-1 secretion, was examined. For this purpose, MCP-1 levels in cell supernatants were determined via MCP-1 ELISA after 24 h of incubation. Under basal conditions, MCP-1 secretion was not affected by flavonoids, except for the highest concentrations of luteolin and kaempferol, which caused a highly significant decrease in MCP-1 concentrations (Figure 5A,C,E,G). Stimulation of HCAEC with IL-1β resulted in a significant increase in MCP-1 levels in cell supernatants compared with unstimulated cells (Figure 5B,D,F,H). Quercetin, kaempferol, and luteolin were able to counteract these increased MCP-1 levels in the cell supernatants in a concentration-dependent manner (Figure 5B,D,F). At higher concentrations, cannflavin A led to a slight reduction in IL-1β-induced MCP-1 secretion, which was, however, much lower than the effects of the other flavonoids (Figure 5H).

### 2.5. Migration of Monocytes toward CM of IL-1β-Stimulated HCAEC Is Prevented by Treatment of HCAEC with Quercetin, Kaempferol, and Luteolin but Not with Cannflavin A

**Figure 6:**
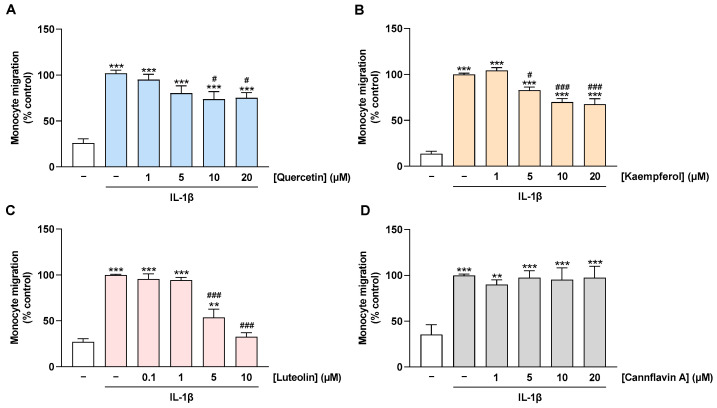
Effect of flavonoids on monocyte migration toward CM of IL-1β-stimulated HCAEC. HCAEC were preincubated with increasing concentrations of quercetin (**A**), kaempferol (**B**), luteolin (**C**), cannflavin A (**D**), or with vehicle for 1 h, followed by the addition of 10 ng/mL IL-1β or its vehicle and subsequent coincubation for 24 h. Then, CMs were collected and migration of THP-1 monocytes toward CM of HCAEC was run for 6 h. Monocyte migration was determined via Boyden chamber assay. Migration of monocytes toward the CM of HCAEC treated with vehicle in combination with IL-1β was set to 100%. Data are presented as means ± SEM of *n* = 10 (five independent experiments, (**A**)) or *n* = 6 (three independent experiments, (**B**–**D**)). ** *p* ≤ 0.01, *** *p* ≤ 0.001 vs. vehicle control (leftmost white bar); # *p* ≤ 0.05, ### *p* ≤ 0.001 vs. IL-1β-stimulated cells; one-way ANOVA with Bonferroni’s post hoc test.

The decrease in MCP-1 levels in culture supernatants of flavonoid-treated cells implies a possible impairment of monocyte migration to the activated endothelium at the functional level. To investigate this issue, a Boyden chamber assay was performed to examine the migration of THP-1 monocytes toward the CM of HCAEC. For this purpose, HCAEC were treated with flavonoids and stimulated with IL-1β for 24 h before the CMs were collected. Thereby, the CM from IL-1β-stimulated HCAEC caused an induction of monocyte migration when compared with the CM from non-stimulated cells (Figure 6A–D). This induction was suppressed by preincubation of HCAEC with quercetin, kaempferol, and luteolin in a concentration-dependent manner (Figure 6A–C). On the other hand, as expected, treatment of HCAEC with cannflavin A showed no effect on the migration of monocytes toward CM from IL-1β-stimulated HCAEC (Figure 6D).

### 2.6. MCP-1 Is an Important Mediator of Monocyte Migration

**Figure 7:**
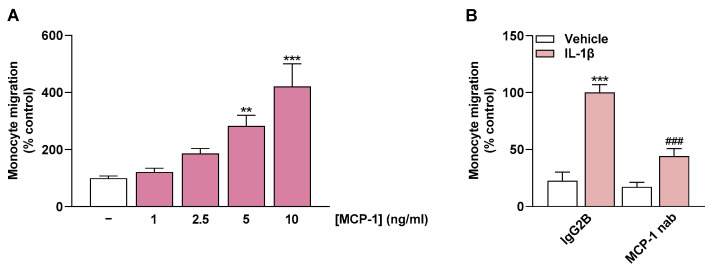
Effect of recombinant MCP-1 (**A**) and a neutralizing antibody (nab) directed against MCP-1 (**B**) on monocyte migration. Migration of THP-1 cells was determined with Boyden chamber assay toward increasing MCP-1 concentrations (**A**) or toward CM from HCAEC (**B**). In (**A**), medium containing recombinant MCP-1 added to the lower chamber was used to induce monocyte migration. In (**B**), CM from HCAEC previously treated with IL-1β or vehicle for 24 h was incubated with MCP-1 antibody (1 µg/mL) or the appropriate isotype control IgG2B (1 µg/mL) for 1 h after removal from the cells. Thereafter, migration of THP-1 monocytes toward CM was initiated. In both settings (**A**,**B**), migration was run for 6 h. Monocyte migration toward vehicle-containing medium (**A**) or toward CM of IL-1β-treated HCAEC containing IgG2B (**B**) were set to 100%. Data are presented as means ± SEM of *n* = 5–6 (**A**) or *n* = 6 (**B**) of three independent experiments each. ** *p* ≤ 0.01, *** *p* ≤ 0.001 vs. vehicle control; one-way ANOVA with Dunnett’s post hoc test (**A**). *** p ≤ 0.001 vs. vehicle control (leftmost white bar); ### *p* ≤ 0.001 vs. IL-1β-stimulated cells; one-way ANOVA with Bonferroni’s post hoc test (**B**).

To relate the flavonoid-dependent impairment of monocyte migration to the MCP-1-lowering property of these compounds, final experiments were performed with recombinant MCP-1 and with an MCP-1-neutralizing antibody. These experiments were designed to demonstrate a general effect of MCP-1 in the experimental migration setup. Thereby, in initial experiments, increasing concentrations of recombinant MCP-1 resulted in a linear increase in monocyte migration (Figure 7A).

In the second part, MCP-1 was neutralized with an MCP-1-neutralizing antibody in the CM of previously IL-1β-treated HCAEC. The CM treated in this manner resulted in significantly less monocyte migration than the CM from IL-1β-stimulated HCAEC incubated with an IgG2B Isotype Control (Figure 7B). This finding highlights the mediating role of MCP-1 in monocyte migration toward CM of IL-1β-treated HCAEC.

## 3. Discussion

In the present study, the effects of the flavonoids quercetin, kaempferol, luteolin, and cannflavin A on MCP-1 expression and secretion in HCAEC were investigated. To this end, IL-1β, one of the most prominent proinflammatory cytokines [32], was used to induce an activated state of HCAEC. Our data show that the flavonols quercetin and kaempferol and the flavone luteolin exert an inhibitory effect on MCP-1 mRNA expression and secretion in stimulated HCAEC. In addition to suppression of MCP-1, migration of human THP-1 monocytes into the CM of IL-1β-stimulated HCAEC was reduced when the latter were coincubated with quercetin, kaempferol, and luteolin. The impairment of monocyte migration by these flavonoids is most likely related to MCP-1 inhibition. This is supported by the fact that, on one hand, migration induced by the CM of IL-1β-stimulated HCAEC was highly significantly inhibited when MCP-1 was previously neutralized in the medium. On the other hand, when recombinant MCP-1 was used as a chemoattractant in the lower Boyden chamber, a clear concentration-dependent induction of monocyte migration was demonstrated.

The functional effects shown here are of particular importance. As outlined earlier, the recruitment of circulating monocytes to inflamed tissue represents one of the first steps in the formation of atherosclerotic lesions, making the suppression of monocyte migration an important target for the prevention and treatment of cardiovascular diseases [33]. As for flavonoids, their direct influence on monocyte migration has been studied so far. Accordingly, quercetin caused inhibition of MCP-1 expression in THP-1 monocytes and decreased migration of these cells [34]. The latter was evident when THP-1 cells seeded on the top of the transwell insert were treated with the flavonoid and MCP-1 was added to the lower chamber as a chemoattractant [34]. Comparable inhibitory effects in this system were also described later for kaempferol by the same group [35]. In the case of luteolin, this flavonoid was shown to reduce IL-4-mediated expression and release of MCP-1 from RAW 264.7 macrophages. Again, treatment of macrophages in the lower chambers of a transwell system with luteolin before IL-4 stimulation resulted in inhibition of migration of THP-1 cells into the lower chamber, with the corresponding inhibitory effect being reversed by the addition of recombinant MCP-1 [36]. Therefore, the flavonoids quercetin, kaempferol, and luteolin appear to be able to inhibit monocyte migration both directly by affecting monocytes or macrophages and indirectly by downregulating MCP-1 in endothelial cells.

Although the present study is the first to describe the inhibition of MCP-1 expression by flavonoids in HCAEC, corresponding effects have also been found in other endothelial cells [37,38,39,40,41,42,43,44,45]. In this context, inhibition of the activation of the transcription factor NF-κB seems to be an important underlying mechanism, as shown for quercetin [37,38,39,40], kaempferol [37,40], and luteolin [41,46]. Other flavonoid-mediated mechanisms associated with MCP-1 downregulation include inhibition of transcription factor AP-1 [38,42], inhibition of Akt phosphorylation [43], and activation of the Nrf2/heme oxygenase-1 pathway [44,45].

It is also noteworthy that cannflavin A, the only prenylated flavonoid tested, did not induce a relevant decrease in MCP-1 as well as monocytic migration. Since inhibition of the proinflammatory NF-κB pathway often requires an antioxidant effect of the respective substance, it would be interesting to perform a comparative analysis with other flavonoids in subsequent studies. Indeed, no inhibitory effect could be demonstrated for cannflavin A in the DPPH (1,1-diphenyl-2-picrylhydrazyl) assay, which is considered a valid and accurate method for evaluating the free radical scavenging activity of antioxidants [47]. On the other hand, quercetin [48], kaempferol [49], and luteolin [50] proved to be active in this test, revealing differences from cannflavin A.

In the present study, partial inhibition of induced MCP-1 formation was demonstrated for quercetin, kaempferol, and luteolin. On the other hand, previous studies have shown that flavonoids inhibit a whole range of proinflammatory mediators in stimulated endothelial cells, including the adhesion molecules vascular cell adhesion molecule-1 (VCAM-1) and intercellular adhesion molecule-1 (ICAM-1) [37,39,40,41,45], the prostaglandin-generating enzyme cyclooxygenase-2 [37], the inducible NO synthase [37], or the macrophage colony-stimulating factor (M-CSF) [40]. A synergistic effect of flavonoids should also be considered, as shown by the combined effect of luteolin and curcumin on TNF-α-enhanced protein levels of VCAM-1 and MCP-1 or the nuclear translocation of NF-κB in endothelial cells [51].

To date, there have been no studies examining cannflavin A in human vascular endothelial cells. In preclinical studies, cannflavin A showed an anti-inflammatory activity via inhibition of cellular prostaglandin E_2_ release from stimulated synovial cells [52] and inhibition of cell-free microsomal prostaglandin E synthase-1 [47]. Although not yet tested, this effect could also have a positive impact on endothelial inflammation. Otherwise, cannflavin A was recently found to have anticancer activity in bladder cancer [53] and neuroprotective properties in Alzheimer’s disease [54], suggesting additional potential clinical benefits of this flavonoid.

It remains to be mentioned, however, that although pharmacological inhibition of MCP-1 has proven successful in preclinical studies, a corresponding translation into clinical trials is still awaited. This appears to be mainly due to the promiscuity of the MCP-1–CCR2 axis, the complex molecular structure of MCP-1 and its receptor, and problems in delivering the drug to the desired tissue [8,16]. For example, in a clinical trial aimed at evaluating the efficacy and safety of the MCP-1 inhibitor bindarit in preventing restenosis after percutaneous coronary intervention [55], the primary endpoint was not met. However, the significant reduction in the in-stent late loss suggested that bindarit likely exerts a beneficial effect on the vessel wall after angioplasty. In another randomized trial, MLN1202, a highly specific humanized monoclonal antibody that interacts with CCR2 and inhibits MCP-1 binding, was investigated [56]. In this study, MLN1202 significantly reduced levels of high-sensitivity C-reactive protein in patients at risk for atherosclerotic cardiovascular disease [56]. This finding was restricted to patients carrying the A/G or G/G single nucleotide polymorphism in the MCP-1 promoter, thus supporting a molecular epidemiological association between these genotypes and an increased risk of atherosclerotic cardiovascular disease. However, the study was limited in size and no phase III studies of this project were reported [8].

Overall, the antioxidant and anti-inflammatory flavonoids quercetin, kaempferol, and luteolin, but not cannflavin A, were found to substantially inhibit MCP-1 expression and secretion in HCAEC and to impair MCP-1-triggered migration of monocytes. These potential antiatherogenic effects should be further pursued preclinically and clinically to provide new agents for the prophylaxis and treatment of atherosclerosis and resulting cardiovascular diseases.

## 4. Materials and Methods

### 4.1. Materials

Cannflavin A (#16342) was purchased from PhytoLab (Vestenbergsgreuth, Germany). Kaempferol (#11852), luteolin (#10004161), and quercetin (#10005169) were bought from Cayman Chemical (Ann Arbor, MI, USA). Calcein-AM (#17783), IL-1β human (#SRP3083) and MTT (3-(4,5-dimethyl-2-thiazolyl)-2,5-diphenyl-2H-tetrazolium bromide; #M2128) were obtained from Sigma-Aldrich (Taufkirchen, Germany). BD Pharmingen^TM^ Recombinant Human MCP-1 (#554620) was purchased from BD Biosciences (Franklin Lakes, NJ, USA). IgG2B Isotype Control (#MAB004; RRID:AB_357346) and CCL2/JE/MCP-1 Antibody (#MAB679; RRID:AB_2071559) were obtained from R&D Systems (Wiesbaden, Germany). WST-1 ((4-[3-(4-iodophenyl)-2-(4-nitrophenyl)-2H-5-tetrazolio]-1,3-benzene disulfonate) was bought from Roche Diagnostics (Mannheim, Germany). Dithiothreitol (DTT) was obtained from Biomol (Hamburg, Germany). Aqua ad iniectabilia was purchased from Braun Melsungen (Melsungen, Germany). Acetic acid and dimethyl sulfoxide (DMSO) were purchased from AppliChem (Darmstadt, Germany). Albumin (IgG-free), crystal violet, and Triton^®^ X-100 were from Carl Roth (Karlsruhe, Germany). Gibco^TM^ penicillin–streptomycin and Gibco^TM^ trypsin–EDTA were from Thermo Fisher Scientific (Schwerte, Germany). Dulbecco’s phosphate-buffered saline (DPBS) and fetal bovine serum (FBS) were purchased from PAN-Biotech (Aidenach, Germany).

### 4.2. Cell Culture

Human coronary artery endothelial cells (HCAEC, #C-12221) were purchased from Promocell (Heidelberg, Germany) and cultured in Endothelial Cell Growth Medium MV2 (#C-22121; Promocell) containing 5% fetal calf serum (FCS), 5 ng/mL recombinant human epidermal growth factor, 10 ng/mL recombinant human basic fibroblast growth factor, 20 ng/mL recombinant human insulin-like growth factor, 0.5 ng/mL recombinant human vascular endothelial growth factor 165, 1 µg/mL ascorbic acid, and 0.2 µg/mL hydrocortisone. The medium was additionally supplemented with 100 U/mL penicillin and 100 μg/mL streptomycin. In the following, this composition is also referred to as “complete growth medium”. On the other hand, “reduced medium” refers to 2% FCS-containing medium supplemented with 100 U/mL penicillin and 100 μg/mL streptomycin but without growth supplements. HCAEC were cultured in a humidified atmosphere at 37 °C and 5% CO_2_.

All HCAEC experiments with test substances were performed in reduced medium after cells were washed with DPBS. Test substances were dissolved in DMSO (quercetin, kaempferol, luteolin, and cannflavin A), aqua ad iniectabilia (IL-1β), DPBS (MCP-1 antibody, IgG2B Isotype Control), or DPBS and 0.5 mg/mL albumin (recombinant human MCP-1). The final concentration of solvents in the incubation media of cells treated with test substances and vehicle did not exceed 0.1% (*v*/*v*) DMSO, 0.1% (*v*/*v*) aqua ad iniectabilia, 0.2% (*v*/*v*) DPBS, or 0.1% (*v*/*v*) DPBS and 0.5 µg/mL albumin. Vehicle-treated cells contained the same amounts and concentrations of solvents as substance-treated cells.

The human monocytic cell line THP-1 (ACC16, RRID:CVCL_0006) was purchased from the German Collection of Microorganisms and Cell Cultures (DSMZ; Braunschweig, Germany). THP-1 cells were cultured in RPMI 1640 with L-Glutamine (#BE12-702F, Lonza Group, Basel, Switzerland) supplemented with 10% FBS, 100 U/mL penicillin, and 100 μg/mL streptomycin. THP-1 cells were cultured in a humidified atmosphere at 37 °C and 5% CO_2_.

### 4.3. Cellular Viability Assays

For analysis of HCAEC cell viability, three different assays were performed. To this end, cells were seeded in 96-well plates at a density of 5000 cells/well and grown for 24 h in complete growth medium. Then, cells were maintained in reduced medium and preincubated with the test compounds or vehicle for 1 h, followed by the addition of 10 ng/mL IL-1β or its vehicle and subsequent coincubation for 24 h. Afterwards, viability was determined.

Colorimetric MTT and WST-1 assays were used to assess the metabolic activity of HCAEC. Thereby, intracellular MTT was reduced to an insoluble formazan via NAD(P)H-dependent cellular oxidoreductase enzymes. In the case of the cell-impermeable WST-1, on the other hand, the reduction occurred outside the cell via electron transport of the plasma membrane. For the MTT assay, MTT solution (3 mg/mL in DPBS, 0.5 mg/mL final concentration in incubates) was added after incubation with the test compounds was complete, and the cells were then incubated with the reagent for an additional 2 h. The supernatant was then removed, the crystals formed were redissolved with DMSO and the absorbance was measured at 570 nm/690 nm using a microplate reader (Infinite F200 Pro Tecan, Tecan Group, Männedorf, Switzerland). For the WST-1 assay, the water-soluble tetrazolium salt WST-1 was added after incubation was complete, followed by an additional incubation period of at least 30 min. Afterwards, the absorbance was measured at 450 nm/690 nm using a microplate reader.

To determine the effects of the test compounds on the number of remaining viable adherent cells, the crystal violet assay was used. Crystal violet is an intercalating dye that binds to cellular proteins and DNA, so that crystal violet staining decreases with cell death and subsequent detachment from cell culture plates. Here, cells were first fixed overnight with ice-cold absolute ethanol. Subsequently, the cells were incubated with 0.1% (*w*/*v*) crystal violet in 10% (*v*/*v*) ethanol for 30 min. Following washing off the excess dye, the stain was resolved with 10% (*v*/*v*) acetic acid and the absorbance was measured at 570 nm using a microplate reader.

### 4.4. Quantitative Reverse Transcriptase Polymerase Chain Reaction (qRT-PCR)

HCAEC were seeded at 200,000 cells/well in 6-well plates and grown in complete growth medium for 24 h. After washing with DPBS, HCAEC were maintained in reduced medium, preincubated with test substances for 1 h, followed by the addition of 10 ng/mL IL-1β or its vehicle and subsequent coincubation for 8 h. Cells were harvested by collecting the cell supernatants and washing with warm DPBS. Cells were then separated with trypsin–EDTA, collected with the cell supernatants, and centrifuged at 200× *g*, 4 °C for 5 min. The cell pellets were again washed with DPBS and centrifuged at 250× *g* and 4 °C for 5 min. Total RNA was isolated from the resulting cell pellet using the RNeasy Mini Kit from Qiagen (Hilden, Germany) according to the manufacturer’s instructions. Total RNA concentrations were measured using the NanoDrop™ OneC Microvolume UV-Vis spectrophotometer (Thermo Fisher Scientific).

For quantification of MCP-1 mRNA, the Applied Biosystems TaqMan^®^ Gene Expression Assay (MCP-1/CCL2: Hs00234140_m1; FAM-MGB) and Applied Biosystems TaqMan^®^ RNA-to-CT™ 1-Step Kit from Thermo Fisher Scientific were used according to the manufacturer’s instructions. Peptidylprolyl isomerase A (PPIA: Hs999904_m1; VIC-MGB) was used as a housekeeping gene to normalize MCP-1 mRNA levels before comparison with respective vehicle controls.

### 4.5. MCP-1 ELISA

HCAEC were seeded in 48-well plates at a density of 50,000 cells/well, grown in complete growth medium for 24 h, and then maintained in reduced medium. After a 1 h preincubation with the flavonoids or vehicle, 10 ng/mL IL-1β or its vehicle were added, followed by a 24 h coincubation. To investigate the effect on basal MCP-1 secretion in a separate experiment, HCAEC were incubated with the flavonoids in the absence of IL-1β for 25 h. After completion of the incubation, the cell supernatants were collected. Supernatants were centrifuged at 1300× *g* and 4 °C for 5 min and collected again. Quantification of MCP-1 in cell culture supernatants was performed according to the instructions of Quantikine^®^ Human CCL2/MCP-1 Immunoassay Kit (R&D Systems).

### 4.6. Monocyte Migration Assay

Monocyte migration was induced with CM from HCAEC. To prepare the CM, endothelial cells were seeded in 48-well plates at a density of 50,000 cells/well and cultured in complete growth medium for 24 h before reduced medium was added. After a 1 h preincubation with the test substances or vehicle, 10 ng/mL IL-1β or its vehicle were added, and following a subsequent coincubation for 24 h, the cell supernatants were collected. The CMs were then centrifuged at 250× *g* at room temperature for 5 min, and the supernatants were collected.

Before starting the migration assay, THP-1 cells were stained with 5 µM calcein-AM in serum-free RPMI-1640 medium for 30 min and then washed with DPBS and maintained in reduced endothelial cell medium.

Migration experiments were performed with a Boyden chamber assay using Falcon^®^ cell culture inserts containing a polyethylene terephthalate membrane with 8 µm pores (#353097; Corning, Corning, NY, USA). In this assay, THP-1 cells seeded onto the inserts must migrate through the membrane with 8 µm pores to the CM of HCAEC in the 24-well companion plate (lower compartment). To this end, 500,000 stained THP-1 cells were added to the upper chamber, and the lower chamber was filled with the CM. After an incubation period of 6 h, the media from the lower chambers were collected and centrifuged at 250× *g* and room temperature for 5 min. Cell pellets were then resuspended in reduced endothelial cell medium containing 2% (*v*/*v*) Triton^®^ X-100, and samples were transferred to a 96-well plate. After 20 min of incubation, fluorescence was measured at 485 nm (emission)/535 nm (excitation) using a microplate reader.

To investigate the effect of recombinant MCP-1 on monocyte migration, the assay was performed as described above but using recombinant MCP-1 in reduced medium instead of CM from HCAEC.

For neutralization experiments, HCAEC were seeded as above, stimulated with IL-1β or vehicle for 24 h, and then the cell culture supernatants were collected. The CMs were incubated with MCP-1 antibody or an IgG2B Isotype Control in a 24-well plate for 1 h before performing the migration assay.

### 4.7. Statistics

All statistical analyses were performed using GraphPad Prism 9.1.0 or a later version (GraphPad Software, San Diego, CA, USA). Comparisons between groups were performed using one-way ANOVA with Dunnett’s post hoc test when all conditions were compared with a vehicle control or using the Bonferroni post hoc test for selected group comparisons.

## Data Availability

Data are available upon reasonable request from the first author.

## Supplementary Materials

The following supporting information can be downloaded at: https://www.mdpi.com/article/10.3390/ijms242216047/s1.

## References

[1] World Health Organization Cardiovascular Diseases (CVDs) Fact Sheet. https://www.who.int/news-room/fact-sheets/detail/cardiovascular-diseases-(cvds).

[2] Ross R. (1999). Atherosclerosis—An inflammatory disease. N. Engl. J. Med..

[3] Otsuka F., Finn A.V., Yazdani S.K., Nakano M., Kolodgie F.D., Virmani R. (2012). The importance of the endothelium in atherothrombosis and coronary stenting. Nat. Rev. Cardiol..

[4] Géraud C., Koch P.-S., Goerdt S. (2014). Vascular niches: Endothelial cells as tissue- and site-specific multifunctional team players in health and disease. JDDG J. Dtsch. Dermatol. Ges..

[5] Noels H., Weber C., Koenen R.R. (2019). Chemokines as Therapeutic Targets in Cardiovascular Disease: The Road Behind, The Road Ahead. Arterioscler. Thromb. Vasc. Biol..

[6] Deshmane S.L., Kremlev S., Amini S., Sawaya B.E. (2009). Monocyte chemoattractant protein-1 (MCP-1): An overview. J. Interferon Cytokine Res..

[7] Singh S., Anshita D., Ravichandiran V. (2021). MCP-1: Function, regulation, and involvement in disease. Int. Immunopharmacol..

[8] Zhang H., Yang K., Chen F., Liu Q., Ni J., Cao W., Hua Y., He F., Liu Z., Li L. (2022). Role of the CCL2-CCR2 axis in cardiovascular disease: Pathogenesis and clinical implications. Front. Immunol..

[9] Navab M., Imes S.S., Hama S.Y., Hough G.P., Ross L.A., Bork R.W., Valente A.J., Berliner J.A., Drinkwater D.C., Laks H. (1991). Monocyte transmigration induced by modification of low density lipoprotein in cocultures of human aortic wall cells is due to induction of monocyte chemotactic protein 1 synthesis and is abolished by high density lipoprotein. J. Clin. Investig..

[10] Van Coillie E., Van Damme J., Opdenakker G. (1999). The MCP/eotaxin subfamily of CC chemokines. Cytokine Growth Factor Rev..

[11] Nelken N.A., Coughlin S.R., Gordon D., Wilcox J.N. (1991). Monocyte chemoattractant protein-1 in human atheromatous plaques. J. Clin. Investig..

[12] Živković L., Asare Y., Bernhagen J., Dichgans M., Georgakis M.K. (2022). Pharmacological Targeting of the CCL2/CCR2 Axis for Atheroprotection: A Meta-Analysis of Preclinical Studies. Arterioscler. Thromb. Vasc. Biol..

[13] Ni W., Egashira K., Kitamoto S., Kataoka C., Koyanagi M., Inoue S., Imaizumi K., Akiyama C., Nishida K., Takeshita A. (2001). New anti-monocyte chemoattractant protein-1 gene therapy attenuates atherosclerosis in apolipoprotein E-knockout mice. Circulation.

[14] Georgakis M.K., van der Laan S.W., Asare Y., Mekke J.M., Haitjema S., Schoneveld A.H., de Jager S.C.A., Nurmohamed N.S., Kroon J., Stroes E.S.G. (2021). Monocyte-Chemoattractant Protein-1 Levels in Human Atherosclerotic Lesions Associate With Plaque Vulnerability. Arterioscler. Thromb. Vasc. Biol..

[15] Georgakis M.K., de Lemos J.A., Ayers C., Wang B., Björkbacka H., Pana T.A., Thorand B., Sun C., Fani L., Malik R. (2021). Association of Circulating Monocyte Chemoattractant Protein-1 Levels With Cardiovascular Mortality: A Meta-analysis of Population-Based Studies. JAMA Cardiol..

[16] Georgakis M.K., Bernhagen J., Heitman L.H., Weber C., Dichgans M. (2022). Targeting the CCL2-CCR2 axis for atheroprotection. Eur. Heart J..

[17] Panche A.N., Diwan A.D., Chandra S.R. (2016). Flavonoids: An overview. J. Nutr. Sci..

[18] Rees A., Dodd G.F., Spencer J.P.E. (2018). The Effects of Flavonoids on Cardiovascular Health: A Review of Human Intervention Trials and Implications for Cerebrovascular Function. Nutrients.

[19] Chen Z., Zhang S.L. (2021). The role of flavonoids in the prevention and management of cardiovascular complications: A narrative review. Ann. Palliat. Med..

[20] Kumar S., Pandey A.K. (2013). Chemistry and biological activities of flavonoids: An overview. Sci. World J..

[21] Dabeek W.M., Marra M.V. (2019). Dietary Quercetin and Kaempferol: Bioavailability and Potential Cardiovascular-Related Bioactivity in Humans. Nutrients.

[22] Papakyriakopoulou P., Velidakis N., Khattab E., Valsami G., Korakianitis I., Kadoglou N.P. (2022). Potential Pharmaceutical Applications of Quercetin in Cardiovascular Diseases. Pharmaceuticals.

[23] Li L., Luo W., Qian Y., Zhu W., Qian J., Li J., Jin Y., Xu X., Liang G. (2019). Luteolin protects against diabetic cardiomyopathy by inhibiting NF-κB-mediated inflammation and activating the Nrf2-mediated antioxidant responses. Phytomedicine.

[24] Wu B., Song H., Fan M., You F., Zhang L., Luo J., Li J., Wang L., Li C., Yuan M. (2020). Luteolin attenuates sepsis-induced myocardial injury by enhancing autophagy in mice. Int. J. Mol. Med..

[25] Erridge S., Mangal N., Salazar O., Pacchetti B., Sodergren M.H. (2020). Cannflavins—From plant to patient: A scoping review. Fitoterapia.

[26] Li X., Sun S., Chen D., Yuan T., Chen Y., Wang D., Fang L., Lu Y., Du G. (2020). Puerarin attenuates the endothelial-mesenchymal transition induced by oxidative stress in human coronary artery endothelial cells through PI3K/AKT pathway. Eur. J. Pharmacol..

[27] Li X., Yuan T., Chen D., Chen Y., Sun S., Wang D., Fang L., Lu Y., Du G. (2018). Cardioprotective Effects of Puerarin-V on Isoproterenol-Induced Myocardial Infarction Mice Is Associated with Regulation of PPAR-γ/NF-κB Pathway. Molecules.

[28] Reddy A.T., Lakshmi S.P., Maruthi Prasad E., Varadacharyulu N.C., Kodidhela L.D. (2020). Epigallocatechin gallate suppresses inflammation in human coronary artery endothelial cells by inhibiting NF-κB. Life Sci..

[29] Ramirez-Sanchez I., Maya L., Ceballos G., Villarreal F. (2010). (−)-epicatechin activation of endothelial cell endothelial nitric oxide synthase, nitric oxide, and related signaling pathways. Hypertension.

[30] Ramírez-Sánchez I., Rodríguez A., Moreno-Ulloa A., Ceballos G., Villarreal F. (2016). (−)-Epicatechin-induced recovery of mitochondria from simulated diabetes: Potential role of endothelial nitric oxide synthase. Diabetes Vasc. Dis. Res..

[31] Pasten C., Olave N.C., Zhou L., Tabengwa E.M., Wolkowicz P.E., Grenett H.E. (2007). Polyphenols downregulate PAI-1 gene expression in cultured human coronary artery endothelial cells: Molecular contributor to cardiovascular protection. Thromb. Res..

[32] Gabay C., Lamacchia C., Palmer G. (2010). IL-1 pathways in inflammation and human diseases. Nat. Rev. Rheumatol..

[33] Gerhardt T., Ley K. (2015). Monocyte trafficking across the vessel wall. Cardiovasc. Res..

[34] Huwait E.A., Saddeek S.Y., Al-Massabi R.F., Almowallad S.J., Pushparaj P.N., Kalamegam G. (2021). Antiatherogenic Effects of Quercetin in the THP-1 Macrophage Model In Vitro, with Insights into Its Signaling Mechanisms Using In Silico Analysis. Front. Pharmacol..

[35] Huwait E., Ayoub M., Karim S. (2022). Investigation of the Molecular Mechanisms Underlying the Antiatherogenic Actions of Kaempferol in Human THP-1 Macrophages. Int. J. Mol. Sci..

[36] Choi H.-J., Choi H.-J., Chung T.-W., Ha K.-T. (2016). Luteolin inhibits recruitment of monocytes and migration of Lewis lung carcinoma cells by suppressing chemokine (C-C motif) ligand 2 expression in tumor-associated macrophage. Biochem. Biophys. Res. Commun..

[37] Crespo I., García-Mediavilla M.V., Gutiérrez B., Sánchez-Campos S., Tuñón M.J., González-Gallego J. (2008). A comparison of the effects of kaempferol and quercetin on cytokine-induced pro-inflammatory status of cultured human endothelial cells. Br. J. Nutr..

[38] Panicker S.R., Sreenivas P., Babu M.S., Karunagaran D., Kartha C.C. (2010). Quercetin attenuates Monocyte Chemoattractant Protein-1 gene expression in glucose primed aortic endothelial cells through NF-κB and AP-1. Pharmacol. Res..

[39] Bhaskar S., Sudhakaran P.R., Helen A. (2016). Quercetin attenuates atherosclerotic inflammation and adhesion molecule expression by modulating TLR-NF-κB signaling pathway. Cell. Immunol..

[40] Calabriso N., Scoditti E., Massaro M., Pellegrino M., Storelli C., Ingrosso I., Giovinazzo G., Carluccio M.A. (2016). Multiple anti-inflammatory and anti-atherosclerotic properties of red wine polyphenolic extracts: Differential role of hydroxycinnamic acids, flavonols and stilbenes on endothelial inflammatory gene expression. Eur. J. Nutr..

[41] Jia Z., Nallasamy P., Liu D., Shah H., Li J.Z., Chitrakar R., Si H., McCormick J., Zhu H., Zhen W. (2015). Luteolin protects against vascular inflammation in mice and TNF-alpha-induced monocyte adhesion to endothelial cells via suppressing IΚBα/NF-κB signaling pathway. J. Nutr. Biochem..

[42] Chang Y.L., Shen J.J., Wung B.S., Cheng J.J., Wang D.L. (2001). Chinese herbal remedy wogonin inhibits monocyte chemotactic protein-1 gene expression in human endothelial cells. Mol. Pharmacol..

[43] Ahn H.Y., Xu Y., Davidge S.T. (2008). Epigallocatechin-3-*O*-gallate inhibits TNFα-induced monocyte chemotactic protein-1 production from vascular endothelial cells. Life Sci..

[44] Zheng Y., Toborek M., Hennig B. (2010). Epigallocatechin gallate-mediated protection against tumor necrosis factor-α-induced monocyte chemoattractant protein-1 expression is heme oxygenase-1 dependent. Metabolism.

[45] Zhang H.P., Zheng F.L., Zhao J.H., Guo D.X., Chen X.L. (2013). Genistein inhibits ox-LDL-induced VCAM-1, ICAM-1 and MCP-1 expression of HUVECs through heme oxygenase-1. Arch. Med. Res..

[46] Huang W.-C., Liou C.-J., Shen S.-C., Hu S., Hsiao C.-Y., Wu S.-J. (2020). Luteolin Attenuates IL-1β-Induced THP-1 Adhesion to ARPE-19 Cells via Suppression of NF-κB and MAPK Pathways. Mediat. Inflamm..

[47] Werz O., Seegers J., Schaible A.M., Weinigel C., Barz D., Koeberle A., Allegrone G., Pollastro F., Zampieri L., Grassi G. (2014). Cannflavins from hemp sprouts, a novel cannabinoid-free hemp food product, target microsomal prostaglandin E_2_ synthase-1 and 5-lipoxygenase. PharmaNutrition.

[48] Murakami Y., Kawata A., Ito S., Katayama T., Fujisawa S. (2015). Radical-scavenging and Anti-inflammatory Activity of Quercetin and Related Compounds and Their Combinations Against RAW264.7 Cells Stimulated with *Porphyromonas gingivalis* Fimbriae. Relationships between Anti-inflammatory Activity and Quantum Chemical Parameters. In Vivo.

[49] Deng S.P., Yang Y.L., Cheng X.X., Li W.R., Cai J.Y. (2019). Synthesis, Spectroscopic Study and Radical Scavenging Activity of Kaempferol Derivatives: Enhanced Water Solubility and Antioxidant Activity. Int. J. Mol. Sci..

[50] Ahmadi S.M., Farhoosh R., Sharif A., Rezaie M. (2020). Structure-Antioxidant Activity Relationships of Luteolin and Catechin. J. Food Sci..

[51] Zhang L., Wang X., Zhang L., Virgous C., Si H. (2019). Combination of curcumin and luteolin synergistically inhibits TNF-α-induced vascular inflammation in human vascular cells and mice. J. Nutr. Biochem..

[52] Barrett M.L., Scutt A.M., Evans F.J. (1986). Cannflavin A and B, prenylated flavones from *Cannabis sativa* L. Experientia.

[53] Tomko A.M., Whynot E.G., Dupré D.J. (2022). Anti-cancer properties of cannflavin A and potential synergistic effects with gemcitabine, cisplatin, and cannabinoids in bladder cancer. J. Cannabis Res..

[54] Eggers C., Fujitani M., Kato R., Smid S. (2019). Novel cannabis flavonoid, cannflavin A displays both a hormetic and neuroprotective profile against amyloid β-mediated neurotoxicity in PC12 cells: Comparison with geranylated flavonoids, mimulone and diplacone. Biochem. Pharmacol..

[55] Colombo A., Basavarajaiah S., Limbruno U., Picchi A., Lettieri C., Valgimigli M., Sciahbasi A., Prati F., Calabresi M., Pierucci D. (2016). A double-blind randomised study to evaluate the efficacy and safety of bindarit in preventing coronary stent restenosis. EuroIntervention.

[56] Gilbert J., Lekstrom-Himes J., Donaldson D., Lee Y., Hu M., Xu J., Wyant T., Davidson M., MLN1202 Study Group (2011). Effect of CC chemokine receptor 2 CCR2 blockade on serum C-reactive protein in individuals at atherosclerotic risk and with a single nucleotide polymorphism of the monocyte chemoattractant protein-1 promoter region. Am. J. Cardiol..

