# Supplementary material for: Effect of Flavonoids on MCP-1 Expression in Human Coronary Artery Endothelial Cells and Impact on MCP-1-Dependent Migration of Human Monocytes
Source: Int J Mol Sci. 2023 Nov 7;24(22):16047. doi: 10.3390/ijms242216047 (PMC10671372; doi:10.3390/ijms242216047)
Supplement: Supplementary file 1 [file ijms-24-16047-s001.zip › ijms-2647811-supplementary.docx]

Supplementary Material

**Effect of Flavonoids on MCP-1 Expression in Human Coronary Artery Endothelial Cells and Impact on MCP-1-Dependent
Migration of Human Monocytes**

**Lea Brüser ^†^, Elisa Teichmann ^†^ and Burkhard Hinz ***

Institute of Pharmacology and Toxicology, Rostock University Medical Center, Schillingallee 70,
18057 Rostock, Germany; lea.brueser@uni-rostock.de (L.B.); elisa.teichmann@web.de (E.T.)

***** Correspondence: burkhard.hinz@med.uni-rostock.de; Tel.: +49-381-494-5770

^†^ These authors contributed equally to this work.

**Supplementary Figure S1**: Effect of flavonoids on metabolic activity of HCAEC under basal or IL-1β-stimulated conditions as determined by the WST-1 assay. Cells were preincubated with increasing concentrations of quercetin (**A**), kaempferol (**B**), luteolin (**C**), cannflavin A (**D**), or vehicle for 1 h, followed by the addition of 10 ng/mL IL-1β or its vehicle and subsequent coincubation for 24 h. Thereafter, metabolic activity was determined via WST-1 assay. Viability values of vehicle-treated cells were set to 100%. Data are presented as means ± SEM of *n*= 9 (**C**,**D**) or *n* = 8–9 (**A**,**B**) of three independent experiments each. ** p* ≤ 0.05, *** p* ≤ 0.01, *** *p*≤ 0.001 vs. vehicle control (leftmost white bar); # *p* ≤ 0.05, ## *p*≤ 0.01, ### *p* ≤ 0.001 vs. IL-1β-stimulated cells; one-way ANOVA with Bonferroni´s post hoc test.
